# A Safety Net Tobacco Use Cessation Resource: Quitline Service Usage, 2019

**DOI:** 10.5888/pcd20.230033

**Published:** 2023-09-28

**Authors:** Sonia M. Tetlow, Lei Zhang, Mateusz Borowiecki, Yoonsang Kim, Andrea S. Gentzke, Teresa W. Wang, Monica E. Cornelius, Nikki A. Hawkins

**Affiliations:** 1 Office on Smoking and Health, National Center for Chronic Disease Prevention and Health Promotion, Centers for Disease Control and Prevention, Atlanta, Georgia; 2 NORC at the University of Chicago, Chicago, Illinois

## Abstract

**Introduction:**

Quitlines are free, accessible evidence-based services that may provide an important resource for people facing barriers to clinical treatment for cessation of tobacco use.

**Methods:**

Using 2019 intake data from the National Quitline Data Warehouse, we examined quitline service usage, stratified by sociodemographic characteristics. Only US quitlines reporting service type data were included (n = 40 [of 51]). Callers (aged ≥12 years) who registered with a quitline, reported current use of a tobacco product, and received at least 1 service comprised the analytic data. Chi-square tests examined differences in quitline services received by participant characteristics.

**Results:**

In 2019, 182,544 people reporting current use of a tobacco product received at least 1 service from a quitline in 39 states and the District of Columbia. Among them, 80.4% had attained less than a college or university degree and 70.4% were uninsured or enrolled in Medicaid or in Medicare (aged <65 years). By educational attainment (aged ≥25 years), receipt of cessation medications ranged from 59.4% of callers with a college or university degree to 65.0% of callers with a high school diploma (*P < .*001). The range by insurance coverage was 59.3% of callers with private insurance to 74.7% of callers with Medicare (aged <65 years) (*P < .*001).

**Conclusion:**

Quitlines served as a resource for low-SES populations in 2019, providing cessation services to many people who may face barriers to clinical cessation treatment. Strengthening and expanding quitlines may help to increase cessation among populations with a disproportionately high prevalence of tobacco product use and improve the health and well-being of people in the US.

**Summary**

**What is already known on this topic?**

People with low socioeconomic status (SES) represent a large proportion of tobacco quitline callers, yet few studies evaluating quitline effectiveness have examined the generalizability of findings to populations with low SES.

**What is added by this study?**

This study examined quitline service usage stratified by participant sociodemographic characteristics and found significant differences in telephone counseling and cessation medications received by caller characteristics, including educational attainment and health insurance coverage, considered proxy indicators of SES.

**What are the implications for public health practice?**

Future research on quitline efficacy should consider the impact of differences in services received on quit outcomes among participant populations. Programs should also confirm practices that ensure the equitable distribution of services among all quitline participants.

## Introduction

Commercial tobacco product use is the leading cause of preventable disease, disability, and death in the US (1). Though significant gains have been made over time in reducing tobacco product use, prevalence remains disproportionately high among some populations, such as people with low socioeconomic status (SES) (2). SES is a multidimensional construct often measured with indicators of income and educational attainment (2). In 2019, 27.0% of US adults with an annual household income of less than $35,000 reported current use of a tobacco product, compared with 15.1% of adults with an annual household income of at least $100,000 (3). Additionally, more than twice as many US adults with 0 to 12 years of education (no diploma) reported current use of a tobacco product in 2019, compared with adults with a college degree (26.4% and 13.1%, respectively) (3). Similar SES disparities also exist among key indicators of cessation, including past-year quit attempts and achieving successful cessation (4). Long-standing policies, practices, and environmental and social conditions have contributed to disparities in the prevalence of tobacco product use and cessation of use among different populations (2,4). These disparities in turn have contributed to people with low SES experiencing disproportionately the adverse health effects of tobacco use (2).

Quitting smoking confers immediate and long-term health benefits (4). Cessation counseling and pharmacotherapy are evidence-based treatments that increase successful smoking cessation, and are particularly effective when used in combination (4). The Patient Protection and Affordable Care Act requires health insurance plans to cover treatment for cessation of tobacco use, including counseling and the 7 cessation medications approved by the Food and Drug Administration, with no cost-sharing (5). Despite this requirement that extends to most private health insurance and Medicaid expansion plans, cost barriers, such as co-payments for cessation treatment, remain (6–8). Such barriers could limit access to cessation treatment disproportionately among insured populations with low income. Because the policy requirement also does not affect cessation treatment access for people without health insurance, low-income uninsured people may experience disproportionately limited access to cessation treatment because of cost barriers.

Quitlines offer effective, evidence-based services for cessation of tobacco use that are easily accessible via a toll-free telephone number (4,9). All US states and the District of Columbia have quitline programs, many of which also provide web and text-based services (10). Although many quitlines have eligibility requirements, such as state residency and annual limits on the number of services received, quitlines provide important cessation services free from many of the barriers inherent in getting treatment in clinical settings (11). Previous studies found that people with low SES represent a large proportion of quitline callers despite a correlation between lower income and lower awareness of quitlines (12–14). Studies also have found a positive association between use of quitline services and cessation among low-SES populations (15,16). However, a 2021 review concluded that evidence for the efficacy of quitlines among low-SES participants is less clear given that few studies evaluating quitline effectiveness considered or reported the generalizability of findings to this population (17). Additionally, a 2022 systematic review concluded that additional research is needed examining whether and to what extent quitline usage differs by population characteristics, including SES (18). This study fills a gap in the literature by examining quitline service usage stratified by participant sociodemographic characteristics, including indicators of SES, to determine whether participant populations access quitline services in equal measure.

## Methods

### Data source and study sample

We used 2019 intake data from the National Quitline Data Warehouse (NQDW), a repository of data collected by quitline programs and reported to the Centers for Disease Control and Prevention (CDC). Intake data are self-reported data collected from quitline participants during registration for services. Quitline programs in all 50 states, the District of Columbia, Guam, and Puerto Rico as well as the nationwide Asian Smokers Quitline report de-identified individual-level intake data to the NQDW. Given variability in the completeness of information reported by individual quitlines, we restricted the study to programs in states and the District of Columbia and excluded 11 programs (Alabama, Arizona, California, Illinois, Minnesota, Mississippi, New Jersey, New York, South Dakota, Tennessee, and West Virginia) that did not report data on their quitline services to the NQDW (n = 40 of 51).

The analytic data included all participants aged 12 years or older who registered with a quitline in 2019, reported current tobacco use, and received at least 1 quitline service. Three states (Alaska, Missouri, and New Mexico) provided telephone counseling only for adults (aged ≥18 years) in 2019 and thus did not report information on adolescents. Assessment of educational attainment was restricted to participants aged 25 years or older. We limited the sample to unique participants who contacted a quitline seeking help with quitting for themselves.

### Measures

The term “tobacco” as used in this article refers to commercial tobacco products and not to tobacco used for ceremonial purposes by some American Indian communities. We assessed tobacco use from responses to 2 intake questions. We defined current tobacco product users as participants who indicated 1) use of a tobacco product (cigarettes, cigars/cigarillos/little cigars, traditional tobacco pipes not including water pipes or hookahs, chewing tobacco, snuff, dip, and/or other tobacco products) in the last 30 days and 2) current use of that tobacco product every day or some days. A participant was considered to have received a quitline service if they received telephone counseling, cessation medications (nicotine patch, lozenge, gum, or the prescription medications varenicline and bupropion), or self-help materials and information from a quitline. We treated service type categories as mutually exclusive except for medications since all quitline programs included in the study had a counseling requirement in place in 2019 for participants to be eligible for medication. Thus, all participants who received cessation medications also received telephone counseling.

Sociodemographic characteristics consisted of sex (male, female), age (12–17, 18–24, 25–44, 45–64, ≥65 years), and educational attainment (0–12 years [no diploma], General Educational Development (GED) certificate, high school diploma, some college or university, college or university degree). We categorized race and ethnicity as Hispanic or Latino, non-Hispanic American Indian or Alaska Native, non-Hispanic Asian, non-Hispanic Black or African American, non-Hispanic Native Hawaiian or Other Pacific Islander, non-Hispanic White, and non-Hispanic other race. We categorized quitline participants who reported more than 1 race as non-Hispanic multiracial.

We identified health insurance coverage from reported insurance type and provider (private, Medicaid, Medicare aged 65 years or older, Medicare aged less than 65 years, military, and uninsured). We determined intention to quit from responses (yes, no) to the question “Do you intend to quit using [tobacco product] within the next 30 days?” We determined pregnancy status from responses (yes, no) to the question “Are you currently pregnant?” We assigned callers to 1 of 4 US census regions (Northeast, Midwest, South, or West) according to the state from which the participant received quitline services. The extent of missing data varied across measures and ranged from 0.3% for sex to 12.3% for health insurance coverage.

### Statistical analysis

We computed descriptive statistics to examine the distribution of current tobacco product use among quitline participants in 2019 overall and stratified by product type and sociodemographic characteristics. We calculated frequency and percentage of current use for each tobacco product type and for use of 2 or more products. We also calculated frequency and percentage of telephone counseling services and cessation medications received overall and by sociodemographic characteristics. Of note, 2 states (Delaware and Maine) that did not collect or report data on cessation medications were excluded from the analysis of medications received. We used χ^2^ tests to examine differences by participant characteristics in telephone counseling services and cessation medications received. Significance was defined as *P < .*05.

Because 6 states (of the 40 in the analytic data) were missing intake data for 1 or more months, we conducted sensitivity analyses by further restricting data for quitline services received to only states that reported 4 complete quarters of 2019 data. We examined whether significant differences detected in cessation counseling and medications received across participant demographic characteristics reflected true differences or were attributable to incomplete data and found only small differences compared with the results of the primary analyses. We performed all analyses by using Stata version 16 (StataCorp LLC) and SAS version 9.4 (SAS Institute, Inc).

## Results

### Quitline participant characteristics

In 2019, 182,544 people aged 12 years or older who reported current use of a tobacco product registered with and received at least 1 service from a quitline in 39 states and the District of Columbia (Table 1). Most quitline callers smoked cigarettes (96.6%) and 4.4% used 2 or more products. Among those who currently used any tobacco product, 61.1% were female and 38.9% male. By age, 0.1% of participants were aged 12 to 17 years, 3.7% were aged 18 to 24 years, 32.6% were aged 25 to 44 years, 48.2% were aged 45 to 64 years, and 15.4% were aged 65 years or older. Over two-thirds (69.7%) identified as non-Hispanic White, 16.5% as non-Hispanic Black or African American, 6.9% as Hispanic or Latino, and 3.2% as non-Hispanic American Indian or Alaska Native. Less than 1.0% of the sample identified as non-Hispanic Asian, non-Hispanic Native Hawaiian or Other Pacific Islander, or non-Hispanic multiracial. More than half (50.9%) of participants resided in the South, 23.4% in the Midwest, 15.5% in the West, and 10.1% in the Northeast. Participants with a high school diploma comprised the largest proportion of the sample (29.0%), followed by those with some college or university (26.6%), a college or university degree (19.6%), 0 to 12 years of education (no diploma) (16.7%), and a GED certificate (8.1%). The largest proportions of quitline participants were either uninsured (28.7%) or enrolled in Medicaid (28.7%), followed by Medicare (13.0% aged <65 years, 11.8% aged ≥65 years), private insurance (17.2%), and military insurance (0.6%). Nearly all participants in the sample expressed an intent to quit within the next 30 days (97.6%), and nearly all participants who identified as female reported that they were not pregnant (98.5%).

**Table 1:** Quitline Service Usage Among Participants (Aged ≥12 Years) Who Reported Current Tobacco^a^ Product Use and Received at Least 1 Service, by Tobacco Product Used and Selected Characteristics — National Quitline Data Warehouse, 2019^b^

Characteristic	Any tobacco product	Cigarettes	Cigars, cigarillos, or little cigars	Smokeless tobacco	Pipe	Other	≥2 Tobacco products
	No. (%)	No. (%)	No. (%)	No. (%)	No. (%)	No. (%)	No. (%)
**Overall^c^**	182,544 (100)	176,418 (96.6)	6,273 (3.4)	7,687 (4.2)	595 (0.3)	194 (0.1)	8,060 (4.4)
**Sex**							
Male	70,819 (38.9)	66,131 (37.6)	3,559 (57.3)	6,865 (89.7)	416 (72.2)	105 (55.3)	5,837 (73.1)
Female	111,235 (61.1)	109,823 (62.4)	2,656 (42.7)	785 (10.3)	160 (27.8)	85 (44.7)	2,147 (26.9)
**Age group, y**							
12–17^d^	164 (0.1)	149 (0.1)	23 (0.4)	21 (0.3)	3 (0.5)	5 (2.6)	34 (0.4)
18–24	6,756 (3.7)	6,462 (3.7)	514 (8.2)	740 (9.6)	81 (13.6)	18 (9.3)	934 (11.6)
25–44	59,553 (32.6)	57,187 (32.4)	2,458 (39.2)	4,266 (55.5)	229 (38.5)	97 (50.0)	4,366 (54.2)
45–64	87,928 (48.2)	85,325 (48.4)	2,542 (40.5)	2,271 (29.5)	195 (32.8)	56 (28.9)	2,358 (29.3)
≥65	28,143 (15.4)	27,295 (15.5)	736 (11.7)	389 (5.1)	87 (14.6)	18 (9.3)	368 (4.6)
**Race and ethnicity**							
American Indian or Alaska Native, NH	5,719 (3.2)	5,428 (3.2)	265 (4.4)	505 (6.8)	55 (9.5)	4 (2.1)	496 (6.3)
Asian, NH	883 (0.5)	852 (0.5)	17 (0.3)	45 (0.6)	3 (0.5)	1 (0.5)	33 (0.4)
Black or African American, NH	29,275 (16.5)	28,502 (16.7)	1,543 (25.4)	238 (3.2)	40 (6.9)	14 (7.3)	1,026 (13.1)
Hispanic or Latino	12,132 (6.9)	11,910 (7.0)	397 (6.5)	349 (4.7)	33 (5.7)	12 (6.3)	523 (6.7)
Native Hawaiian or Other Pacific Islander, NH	442 (0.2)	432 (0.3)	15 (0.2)	24 (0.3)	3 (0.5)	0	29 (0.4)
Multiracial^e^, NH	1,554 (0.9)	1,491 (0.9)	96 (1.6)	69 (0.9)	13 (2.3)	13 (6.8)	110 (1.4)
White, NH	123,448 (69.7)	119,011 (69.6)	3,562 (58.6)	6,114 (81.8)	417 (72.3)	143 (74.9)	5,420 (69.3)
Other, NH	3,611 (2.0)	3,488 (2.0)	187 (3.1)	126 (1.7)	13 (2.3)	4 (2.1)	189 (2.4)
**US Census region^f^**							
Northeast	18,500 (10.1)	17,967 (10.2)	609 (9.7)	428 (5.6)	51 (8.6)	29 (14.9)	545 (6.8)
Midwest	42,728 (23.4)	41,378 (23.5)	1,645 (26.2)	1,319 (17.2)	123 (20.7)	62 (32.0)	1,688 (20.9)
South	92,989 (50.9)	89,964 (51.0)	3,068 (48.9)	4,247 (55.2)	269 (45.2)	8 (4.1)	4,280 (53.1)
West	28,327 (15.5)	27,109 (15.4)	951 (15.2)	1,693 (22.0)	152 (25.5)	95 (49.0)	1,547 (19.2)
**Education^g^**							
0–12 years (no diploma)	25,461 (16.7)	24,965 (16.9)	866 (16.7)	703 (12.7)	78 (16.7)	18 (12.6)	1,098 (17.4)
General Educational Development certificate	12,350 (8.1)	12,067 (8.2)	444 (8.5)	539 (9.8)	44 (9.4)	11 (7.7)	702 (11.1)
High school diploma	44,242 (29.0)	42,997 (29.1)	1,378 (26.5)	1,615 (29.3)	123 (26.3)	36 (25.2)	1,785 (28.4)
Some college or university	40,476 (26.6)	39,082 (26.4)	1,522 (29.3)	1,525 (27.6)	133 (28.4)	50 (35.0)	1,714 (27.2)
College or university degree	29,856 (19.6)	28,679 (19.4)	987 (19.0)	1,139 (20.6)	90 (19.2)	28 (19.6)	997 (15.8)
**Health insurance coverage**							
Private insurance	26,929 (17.2)	25,457 (16.8)	895 (16.7)	1,658 (26.9)	80 (15.9)	43 (26.9)	1,126 (16.6)
Medicaid	44,859 (28.7)	43,781 (28.9)	1,706 (31.9)	1,354 (22.0)	167 (33.3)	51 (31.9)	2,055 (30.3)
Medicare (aged ≥65 y)	18,514 (11.8)	17,963 (11.9)	444 (8.3)	267 (4.3)	54 (10.8)	10 (6.3)	214 (3.2)
Medicare (aged <65 y)	20,387 (13.0)	19,813 (13.1)	745 (13.9)	474 (7.7)	55 (11.0)	18 (11.3)	682 (10.0)
Military	892 (0.6)	822 (0.5)	33 (0.6)	89 (1.4)	6 (1.2)	3 (1.9)	57 (0.8)
Uninsured	44,887 (28.7)	43,709 (28.8)	1,525 (28.5)	2,314 (37.6)	140 (27.9)	35 (21.9)	2,654 (39.1)
**Intent to quit within the next 30 days^h^**							
Yes	136,941 (97.6)	133,041 (97.7)	4,384 (97.0)	4,615 (97.0)	393 (96.6)	125 (93.3)	5,260 (97.9)
No	3,320 (2.4)	3,136 (2.3)	136 (3.0)	142 (3.0)	14 (3.4)	9 (6.7)	112 (2.1)
**Pregnancy status^i^**							
Pregnant	1,720 (1.5)	1,686 (1.5)	63 (2.4)	18 (2.3)	4 (2.5)	3 (3.5)	51 (2.4)
Not pregnant	109,515 (98.5)	108,137 (98.5)	2,593 (97.6)	767 (97.7)	156 (97.5)	82 (96.5)	2,096 (97.6)

Abbreviation: NH, non-Hispanic.

a The term “tobacco” as used in this article refers to commercial tobacco products and not to tobacco used for medicinal and spiritual purposes by some American Indian communities.

b Quitline participants who reported any tobacco product use “every day” or “some days” and received at least 1 quitline services from the states that submitted data on service type. States that did not report service type data were excluded: Alabama, Arizona, California, Illinois, Minnesota, Mississippi, New Jersey, New York, South Dakota, Tennessee, and West Virginia.

c The percentages of tobacco use by product type sum to >100% because some participants reported use of multiple products.

d Three states (Alaska, Missouri, and New Mexico) provided telephone counseling only for adults (aged ≥18 years) in 2019 and did not report information on adolescents.

e Participants who reported more than 1 race.

f Study quitline programs by US Census regions are Northeast: Connecticut, Maine, Massachusetts, New Hampshire, Pennsylvania, Rhode Island, Vermont; Midwest: Indiana, Iowa, Kansas, Michigan, Missouri, Nebraska, North Dakota, Ohio, Wisconsin; South: Arkansas, Delaware, District of Columbia, Florida, Georgia, Kentucky, Louisiana, Maryland, North Carolina, Oklahoma, South Carolina, Texas, Virginia; and West: Alaska, Colorado, Hawaii, Idaho, Montana, New Mexico, Nevada, Oregon, Utah, Washington, and Wyoming.

g Results are among adults aged ≥25 years.

h Intention to quit was assessed by participants’ intention to quit each type of tobacco product in the next 30 days.

i Only female participants are included in pregnancy status.

### Counseling received

Overall, 88.1% of participants who currently used any tobacco product received telephone counseling: 88.5% among females and 87.5% among males (*P < .*001) (Table 2). By age, 92.7% of participants aged 12 to 17 years, 78.3% of participants aged 18 to 24 years, 82.6% of participants aged 25 to 44 years, 90.8% of participants aged 45 to 64 years, and 93.8% of participants aged 65 years or older received counseling (*P < .*001). By race and ethnicity, 100% of multiracial non-Hispanic, 94.6% of non-Hispanic Black or African American, 91.6% of non-Hispanic Native Hawaiian or Other Pacific Islander, 90.4% of other non-Hispanic, 88.6% of Hispanic or Latino, 87.3% of non-Hispanic White, 85.2% of non-Hispanic Asian, and 68.8% of non-Hispanic American Indian or Alaska Native callers received counseling (*P < .*001). Receipt of telephone counseling ranged geographically from 78.0% of callers residing in the South to 98.9% of callers in the West (*P < .*001). By educational attainment, 95.5% of callers with 0 to 12 years of education (no diploma), 92.1% of callers with a GED certificate, 94.6% of callers with a high school diploma, 92.0% of callers with some college or university, and 91.4% of callers with a college or university degree received telephone counseling (*P < .*001). Larger proportions of quitline participants with Medicaid (93.1%), Medicare and aged 65 years or older (94.1%), and Medicare and aged less than 65 years (93.6%) received counseling as compared with participants with private (84.5%) or military (77.1%) insurance, or participants who were uninsured (81.5%) (*P* < .001). Larger proportions of participants who indicated an intention to quit within 30 days (94.4%) versus no intention to quit (92.7%) received counseling (*P* < .001). There was little difference in the receipt of counseling between callers who were pregnant (88.4%) and those who were not (88.5%).

**Table 2:** Receipt of Telephone Counseling and Cessation Medications Among Quitline Participants (Aged ≥12 Years) Who Reported Current Tobacco^a^ Product Use and Received at Least 1 Service, by Selected Characteristics — National Quitline Data Warehouse, 2019

Characteristic	Telephone counseling only (n = 182,544)^b^		Cessation medications^c^ (n = 176,444)^d^	
	%	*P* value^e^	%	*P* value^e^
**Overall**	88.1	NA	65.2	NA
**Sex**				
Male	87.5	<.001	65.6	.002
Female	88.5		64.9	
**Age group, y**				
12–17^f^	92.7	<.001	0	<.001
18–24	78.3		56.7	
25–44	82.6		61.9	
45–64	90.8		66.7	
≥65	93.8		69.9	
**Race and ethnicity**				
American Indian or Alaska Native, NH	68.8	<.001	70.8	<.001
Asian, NH	85.2		55.3	
Black or African American, NH	94.6		67.3	
Hispanic or Latino	88.6		60.7	
Multiracial^g^, NH	100.0		71.6	
Native Hawaiian or Other Pacific Islander, NH	91.6		59.3	
White, NH	87.3		65.3	
Other, NH	90.4		64.0	
**US Census region^h^**				
Northeast	98.8	<.001	72.8	<.001
Midwest	98.4		66.5	
South	78.0		60.3	
West	98.9		74.6	
**Education^i^**				
0–12 years (no diploma)	95.5	<.001	63.1	<.001
General Educational Development certificate	92.1		59.5	
High school diploma	94.6		65.0	
Some college or university	92.0		61.2	
College or university degree	91.4		59.4	
**Health insurance coverage**				
Private insurance	84.5	<.001	59.3	<.001
Medicaid	93.1		63.1	
Medicare (aged ≥65 y)	94.1		73.0	
Medicare (aged <65 y)	93.6		74.7	
Military	77.1		68.2	
Uninsured	81.5		67.0	
**Intent to quit within the next 30 days^j^**				
Yes	94.4	<.001	64.9	<.001
No	92.7		55.2	
**Pregnancy status^k^**				
Pregnant	88.4	.85	6.6	<.001
Not Pregnant	88.5		65.8	

Abbreviations: NA, not applicable; NH, non-Hispanic.

a The term “tobacco” as used in this article refers to commercial tobacco products and not to tobacco used for medicinal and spiritual purposes by some American Indian communities.

b States that did not report service type data were excluded: Alabama, Arizona, California, Illinois, Minnesota, Mississippi, New Jersey, New York, South Dakota, Tennessee, and West Virginia.

c All participants who received cessation medications also received telephone counseling in accordance with counseling requirements in place in 2019 among the quitline programs included in the study.

d Two additional states that did not report cessation medication data were excluded: Delaware and Maine.

e Chi-square test *P* values testing differences in telephone counseling services and cessation medications received by participant characteristics.

f Three states (Alaska, Missouri, and New Mexico) provided telephone counseling only for adults (aged ≥18 years) in 2019 and did not report information on adolescents.

g Participants who reported more than 1 race.

h Study quitline programs by US Census regions are Northeast: Connecticut, Maine, Massachusetts, New Hampshire, Pennsylvania, Rhode Island, Vermont; Midwest: Indiana, Iowa, Kansas, Michigan, Missouri, Nebraska, North Dakota, Ohio, Wisconsin; South: Arkansas, Delaware, District of Columbia, Florida, Georgia, Kentucky, Louisiana, Maryland, North Carolina, Oklahoma, South Carolina, Texas, Virginia; and West: Alaska, Colorado, Hawaii, Idaho, Montana, New Mexico, Nevada, Oregon, Utah, Washington, and Wyoming.

i Results are among adults aged ≥25 years.

j Intention to quit was assessed by participants’ intention to quit any type of tobacco product in the next 30 days.

k Only female participants are included in pregnancy status.

### Cessation medications received

Among quitlines that reported medication data (n = 38 of 40), 65.2% of callers overall received cessation medications in addition to counseling (Table 2), including 64.9% among female and 65.6% among male participants (*P* = .002). The proportions of callers who received medications increased with age from 0 participants aged 12 to 17 years, 56.7% of participants aged 18 to 24 years, 61.9% of participants aged 25 to 44 years, 66.7% of participants aged 45 to 64 years, to 69.9% of participants aged 65 years or older (*P* < .001). By race and ethnicity, 71.6% of multiracial non-Hispanic, 70.8% of non-Hispanic American Indian or Alaska Native, 67.3% of non-Hispanic Black or African American, 65.3% of non-Hispanic White, 64.0% of other non-Hispanic, 60.7% of Hispanic or Latino, 59.3% of non-Hispanic Native Hawaiian or Other Pacific Islander, and 55.3% of non-Hispanic Asian callers received cessation medications (*P* < .001). By census region, receipt of medications ranged from 60.3% of callers in the South to 74.6% of callers in the West (*P* < .001). By educational attainment, 63.1% of callers with 0 to 12 years of education (no diploma), 59.5% of callers with a GED certificate, 65.0% of callers with a high school diploma, 61.2% of callers with some college or university, and 59.4% of callers with a college or university degree received cessation medications (*P* < .001). Larger proportions of quitline participants with Medicare and aged less than 65 years (74.7%) and Medicare and aged 65 or older (73.0%) received cessation medications as compared with participants with Medicaid (63.1%), private (59.3%) or military (68.2%) insurance, or participants who were uninsured (67.0%) (*P* < .001). A larger proportion of participants who indicated an intention to quit within 30 days (64.9%) received cessation medications compared with those who indicated no intention to quit (55.2%) (*P* < .001). A significantly smaller proportion of callers who were pregnant received medications compared with those who were not (6.6% and 65.8%, respectively; *P* < .001).

## Discussion

Our study results provide an overview of the characteristics of quitline participants who currently used 1 or more tobacco products, registered with a quitline, and received at least 1 cessation service in 2019. Although data on participant income are not reported to the NQDW, low educational attainment, lack of health insurance, enrollment in Medicaid, and enrollment in Medicare (aged <65 years) were used as proxy indicators of low SES (2,19,20). Notably, 80.4% of quitline registrants who received a service had attained less than a college or university degree and 70.4% were uninsured or enrolled in Medicaid or in Medicare (aged <65 years). Both findings indicate that low SES populations made up the majority of callers using quitlines as a cessation resource.

This study also provides an analysis of quitline services received (telephone counseling and cessation medications) by participant sociodemographic characteristics. Results show that telephone counseling and cessation medications were provided to larger proportions of callers with indicators of low versus high SES. Given that quitline treatment reach is low overall among persons who smoke (less than 1%) (10), and lower income is correlated with lower awareness of quitlines (14), increasing awareness of quitlines among people with low SES may further expand their reach and impact in supporting cessation among this population with a high prevalence of tobacco use.

Our findings also reveal some differences in quitline service usage. Overall, the proportion of quitline registrants who received cessation medications was nearly 23 percentage points lower than the proportion that received telephone counseling only. Since the combination of cessation counseling and medication is more effective in increasing successful quitting than either intervention alone (4), this finding suggests that quitline services may be further enhanced if cessation medications were provided proportionate to counseling. Quitline capacity and availability of services is primarily determined by funding, which varies significantly across states (10,21–23). Some states are therefore able to provide more cessation medications or counseling sessions to participants while others implement greater eligibility restrictions such as readiness to quit requirements or insurance requirements that limit service provision among participants who have insurance with cessation benefits (21,22). Although most quitline registrants resided in the South, callers from this region received counseling or medications from quitlines at significantly lower levels than callers from other regions. It is not entirely clear what caused this disparity, but state-determined differences in funding allocated to the programs may have played a role (23). In addition to differences in state funding, some quitlines have obtained approval from the Centers for Medicare and Medicaid Services to claim federal funding at the 50% matching rate for quitline counseling provided to Medicaid beneficiaries (24,25). This supplemental funding for counseling versus cessation medications may have contributed to the difference between receipt of the 2 services among callers enrolled in Medicaid. Additionally, since expanded Medicaid provides more comprehensive cessation coverage than traditional Medicaid (5), this may have contributed to regional disparities because the South contained the largest proportion of states that had not expanded Medicaid in 2019 (26).

Young adults aged 18 to 24 years represented less than 4.0% of quitline participants that used any tobacco product but over 9.0% of participants that used “other” tobacco products, which could reflect the higher use of e-cigarettes among this age group (3). Significantly smaller proportions of young adult quitline participants received counseling and cessation medications than older quitline participants. Because previous research indicates that young adults are both interested in and attempt to quit at similar or higher levels than older age groups (27), our results suggest that quitlines could be further leveraged to reach and provide cessation support for younger populations. Promising practices to expand reach among younger populations include offering digital quitline services, such as web-based resources and interactive tools, and tailored text messaging programs (28). The low proportions of adolescents aged 12 to17 years and pregnant callers who received cessation medications reflect clinical recommendations for these populations (29,30).

Significant differences also emerged in the proportions of counseling or cessation medications received by race and ethnicity. For example, the proportion of non-Hispanic American Indian or Alaska Native participants who received counseling was 18.5 percentage points less than that of non-Hispanic White participants, yet the proportion who received cessation medications was 5.5 percentage points greater. Multiple factors, such as participant treatment preferences, could contribute to such differences. However, this finding could also suggest the need for further examination to understand how these differences emerged and to ensure the equitable distribution of cessation services among all racial and ethnic groups.

### Limitations

Our study provides important insight into quitline service usage by participant characteristics, including indicators of low SES. Results should be considered within the context of a few limitations. First, the results are not nationally generalizable as 11 states did not report data on their quitline services and were excluded from the analyses. Two additional states that did not report data on cessation medications also were excluded from the analysis of medications received. Moreover, as NQDW intake data contain information on participants who contacted quitlines by telephone, our findings may not be generalizable to populations that access quitline services through other modalities, such as web-based or text messaging–based programs. Second, some states were missing 1 or more months of data, and some participants did not respond to every intake question. The extent of missing data varied across measures and ranged from 0.3% for sex to 12.3% for health insurance coverage. For most of the sample, the nonresponses were distributed randomly and should not affect our conclusions. Additionally, sensitivity analyses restricted to data from quitlines that reported complete 2019 intake data revealed only small differences compared with the results of the primary analyses. Third, 3 states (Alaska, Missouri, and New Mexico) provided telephone counseling only for adults (aged ≥18 years) in 2019, and thus did not report information on adolescents. Fourth, some differences in the receipt of services that were significant may not reflect meaningful differences given the large sample size. Lastly, this study relies on administrative data and may be subject to measurement error given variability in the collection and reporting of intake data among quitline programs.

### Conclusion

Addressing commercial tobacco product use is critical to reducing preventable disease, disability, and death in the US. Public health programs can address disparities in tobacco-related illness and death through targeted efforts to increase cessation among populations with disproportionately high prevalences of tobacco product use. Although disparities in tobacco product use persist among groups defined by characteristics such as race and ethnicity and education, low SES is often a cross-cutting characteristic among these different groups (2). Our study found that quitlines primarily served as a resource for low-SES populations in 2019, providing cessation services to many people who are most in need of support for quitting and may face barriers to clinical cessation treatment. Future research can further examine the association between quitline services received and cessation outcomes among low-SES populations. Quitlines could be further leveraged to support cessation among people with low SES by increasing awareness of the availability of these resources among this population. Programs also could confirm that internal practices ensure the equitable distribution of services among all quitline participants. Strengthening and expanding quitlines may help to increase cessation of tobacco use and improve the health and well-being of people in the US.
